# Implicit Motives, Laterality, Sports Participation and Competition in Gymnasts

**DOI:** 10.3389/fpsyg.2020.00900

**Published:** 2020-05-26

**Authors:** Lisa-Marie Schütz, Oliver C. Schultheiss

**Affiliations:** ^1^Institute of Sports and Sports Sciences, Heidelberg University, Heidelberg, Germany; ^2^Department of Psychology, Friedrich–Alexander University Erlangen–Nürnberg, Erlangen, Germany

**Keywords:** implicit motives, performance, gymnasts, competition, sports participation

## Abstract

The implicit motivational needs for power, achievement, and affiliation are relevant for sports performance. Due to their hypothesized association with functions of the right hemisphere (McClelland, 1986), they may influence lateralized perceptual and motor processes. And due to their interactions with motive-specific incentives, they may influence performance conditional on the presence of suitable incentives. This preregistered study, conducted mostly online, examines motivational needs using a standard picture-story exercise (PSE) and their associations with indicators of perceptual and motor laterality and sports performance in gymnasts (*N* = 67). Further it explores how implicit motives interact with suitable motivational incentives in the prediction of sports performance. Results partly confirm a link between indicators of cerebral rightward laterality and implicit motives: the implicit affiliation and achievement motives are positively associated with an indicator of emotional-perceptional laterality (chimeric-faces task), but not with an indicator of motor laterality (turning bias). Moreover, the implicit achievement motive was positively correlated with training hours. The implicit affiliation motive was negatively associated with the highest attained competition level. The presence of achievement incentives (perceived control, failure) and affiliation incentives (training together or alone) did not interact with corresponding motives to predict sports performance.

## Introduction

Implicit motives are non-consciously operating, affect-based dispositions (Schultheiss and Köllner, in press). In contrast, explicit motives are individuals’ beliefs about their motivational needs and are often linked to conscious goals (McClelland et al., 1989). Current research on implicit and explicit motives mostly focuses on three motives: the need for power, affiliation, and achievement (Schultheiss and Köllner, in press). People with a high need for power (n Power) enjoy having an impact on others and try to avoid losing impact or being in a submissive position. People with a strong need for affiliation (n Affiliation) enjoy close and harmonious relationships. People with a strong need for achievement (n Achievement) enjoy the mastery of challenging tasks and try to meet a standard of excellence (Atkinson, 1957; Schultheiss, 2008).

All three motives are highly relevant to sports because they sustain spontaneous behavioral tendencies, such as exercise effort over an extended period of time (cf. McClelland, 1980). In sports, people experience many achievement incentives such as mastery of challenges, competitions (as a means of gauging one’s own skill level), time pressure, testing the limits, and improving personal skills over time. Social incentives include others’ expectations and rewards for good athletic achievements such as a good grade in physical education, praise from teammates, prestige, and high social rank, or money. In the presence of achievement incentives, a high n Achievement is associated with an extended temporal scope of competitive athletic training, sporting success, and sports participation (Krug and Kuhl, 2006; Gröpel et al., 2016, Study 2).

Furthermore, perceived autonomy is hypothesized to play an important role for highly achievement-motivated people. The psychological core of achievement motivation is the ability of an athlete to gain satisfaction from the autonomous mastery of challenging tasks. We therefore assume that achievement-motivated athletes do not like being told what to do because they have been rewarded in their childhood only for autonomous task mastery, but not for mastering tasks under the guidance of others (McClelland et al., 1953; Schultheiss and Brunstein, 2005; see Schultheiss, 2008). The achievement motive drives performance only in situations in which a person can autonomously determine her or his goals. We therefore expect achievement-motivated athletes to require autonomy with regards to their goals and training opportunities in order to be successful. In addition, the experience of failure may have an impact on people’s performance, depending on their implicit achievement motive (Brunstein and Maier, 2005). People high in the implicit achievement motive are more likely to be ready to reach their own standard of excellence or to restore it after failure than people low in this motive.

The affiliation motive can impact the performance of athletes as well. A person’s n Affiliation can be stimulated by the interaction with other group members when teams are reorganized or when new athletes, who may become future friends, are added to the team (Sorrentino and Sheppard, 1978; Wegner et al., 2014). Thus, to the extent that training also entails seeing one’s team mates more frequently, n Affiliation is likely to positively predict the amount of (team) training athletes engage in.

Finally, the power motive can impact performance too. A football player, for example, might perform well and train hard to become the leader of a team. Athletes can also be seen as role models and admired by others. The desire for fame through sporting performance can also be rooted in the power motive (Winter, 1994). Athletes who want to win in a competition, in order to be better than their opponents and thus to have an influence on them and the audience, often have high power motives. These athletes see the competition as a confrontation with their opponents and want to maintain their superiority over them (Krug and Kuhl, 2006; see also Schultheiss and Rohde, 2002).

Although implicit motives are assumed to represent genuine motivational dispositions that direct and energize behavior in response to presently perceived or imagined cues, research indicates that under certain circumstances their enactment benefits from being well-aligned with the explicit beliefs a person holds about her or his motivational needs – that is, when implicit and explicit motives are congruent (e.g., Brunstein and Maier, 2005). Positive effects of motivational congruence have been documented for task performance (Brunstein and Maier, 2005), but also for emotional well-being (Brunstein et al., 1998; Hofer and Busch, 2013; Roch et al., 2017), volitional strength (Gröpel and Kehr, 2014), and identity development (Hofer et al., 2006). We therefore explored whether the congruence between a person’s implicit motive and her or his self-attributed motive in the same domain (e.g., achievement) predicts outcomes related to sports performance above and beyond a main effect of the implicit motive.

Many forms of cognition and behavior are lateralized, and the association between aspects of laterality and implicit motives are a central aspect of the present research. The most easily observable type of lateralization is handedness, with about 90% of the population preferring the right hand for executing key activities such as writing or throwing (e.g., Peters et al., 2006). In sports, rotations, and thus lateralized behavior, play an important role. Humans show a tendency to preferably rotate in one direction (typically to the left; e.g., Yazgan et al., 1996), an effect that is assumed to reflect a dominance of dopaminergic neurotransmission in the contralateral hemisphere. Athletes, for example, choose which direction to turn between a right or left way pirouette, or which foot to use when starting a floor routine (Loffing et al., 2016). In gymnastics, rotations around the vertical axis (pirouettes, screws), the sagittal horizontal axis (cartwheel), and the frontal horizontal axis (somersault) are common. Here, too, rotations can go leftward or rightward. Thus, many of our daily and sports movements are rotations and thus represent lateralized behavior (Güllich and Krüger, 2013; Heinen et al., 2016).

However, laterality effects are not limited to motor behavior or sports performance. Research suggests that emotion and motivation may be lateralized, too. Recent reviews (e.g., McGilchrist, 2009; Gainotti, 2012) suggest that unconscious processing of emotional information preferentially engages a subcortical route of the right hemisphere. Further, a dominance of the right hemisphere can also be observed in automatic behavior such as spontaneous facial expressions, physical motion, imagination, and dreaming. Patients with unilateral brain damage in the right hemisphere show impairments in emotional, facial, and vocal expression and particularly the affective components of language. This is consistent with the hypothesis that the right hemisphere is specialized for decoding and expressing emotion and automatic, unconscious processing more generally (see Gainotti, 2012). Other research could show that the right hemisphere is more involved in imagination, recognizing emotions, fantasy, non-verbal, and visual-spatial tasks (Sperry, 1984) – during fantasizing the right hemisphere seems to be more activated.

These reviews and observations are compatible with earlier speculations by McClelland (1986), who assumed that implicit motivational needs for power, achievement, and affiliation are associated with functions of the right hemisphere. He hypothesized that because implicit motives are expressed and measured in the imaginative stories people write in response to pictures of ambiguous social situations (a procedure called Picture Story Exercise, or PSE), they likely reflect a strong influence of right-hemispheric processes. He contrasted this with people’s responses to well-defined questionnaire items, for which he postulated a stronger left-hemispheric contribution. McClelland’s hypothesis thus suggests that there may be a link between implicit motives and other cognitive and behavioral indicators of cerebral rightward laterality.

More recent studies also suggest parallels between motives and functions of the right hemisphere. First, several studies could show a link between the implicit achievement and power motive and cardiovascular responses (McClelland, 1989; Brunstein and Schmitt, 2010; Mazeres et al., 2019). Brain control over cardiovascular responses is lateralized, with the right hemisphere being dominant in sympathetically mediated control of the human myocardium (Wittling, 1997a; Wittling et al., 1998).

Second, other studies document an involvement of implicit motives in the perception of facial expressions of emotion; that is, for the implicit power motive in detecting emotional expressions (Donhauser et al., 2015), for the implicit affiliation and power motive attentional orienting toward and away from emotional expressions (Schultheiss and Hale, 2007), and affective evaluations of such expressions for all three implicit motives (Rösch et al., 2013). The implicit affiliation and power motive have also been shown to influence how strongly individuals express emotion in the face (e.g., Fodor and Wick, 2009; Kordik et al., 2012). The right hemisphere appears to take the lead when it comes to evaluating (e.g., Bourne, 2010) and expressing emotions in the face (Borod, 1993; Dimberg and Petterson, 2000).

Third, the implicit power motive influences the release of steroid hormones, such as testosterone, estradiol, and cortisol, which are involved in regulating states and behaviors related to dominance, affiliation, and stress (Schultheiss, 2013). Brain control over hormones appears to be lateralized to the right hemisphere, which is capable of eliciting much stronger hormonal responses than the left (Wittling, 1997b; Lueken et al., 2009). Taken together, these parallels between the functions of implicit motives and the functions the right hemisphere specializes in link implicit motives and right hemispheric activity, as hypothesized by McClelland (1986) very likely.

To sum up, the present research investigates three questions. Firstly, it examines if implicit motivational needs for power, achievement, and affiliation are associated with cognitive and behavioral indicators of brain laterality. Secondly, it examines if implicit motives predict sports performance. Thirdly, it explores whether the link between motives and sports performance is moderated by motive-specific incentives and congruence with explicit motives.

Thus, based on McClelland’s (1986) conjecture, our *first preregistered hypothesis* states that all three implicit motives are associated with cognitive-behavioral indicators of rightward brain laterality. We expect perceptual (chimeric faces task) and motor (turning bias) indicators for right hemispheric laterality to be associated with higher motive levels.

Our *second preregistered hypothesis* states that implicit motives (achievement, power, affiliation) are associated with sports performance (hours of training, highest competition level). More specifically, because n Achievement appears to be an indispensable requirement for high sports performance (Krug and Kuhl, 2006, pp. 126–138) we argue that the implicit achievement motive positively correlates with the hours of training and the highest competition level. Because the published literature on the relationships between implicit power and affiliation motives and sports performance is currently sparse, our analyses regarding these motives are exploratory. In addition, based on the documented beneficial effects of congruent explicit goals and implicit motives on self-control (e.g., Gröpel and Kehr, 2014) and performance (Brunstein and Maier, 2005), we also explored to what extent motivational congruence, operationalized as the interaction between an implicit and an explicit motive within the same motivational domain, predict sports performance above and beyond the hypothesized main effects of motives.

Further, our *third preregistered hypothesis*, which is based on previously reviewed arguments and findings regarding interactions between implicit motives and relevant incentives, states that depending on the presence of suitable achievement and affiliation incentives, implicit motives contribute to better sports performance.

## Materials and Methods

### Participants

All participants were informed about the procedure of the study in written form. Participants gave their written informed consent before participating voluntarily in the study. A total of 67 gymnasts (38 women), aged 18–55 years (*M* = 26.52, *SD* = 7.78, *IQR* = 6.00) and residing in Germany participated in this study. All participants currently participated in age-appropriate competitions in international, national, regional, and county leagues such as the German national league or national senior competitions. Data collection was conducted between August 2017 and June 2018. Sample size was limited by the availability of individuals eligible and willing to be tested.

### Design

The study had a cross-sectional design. Criterion variables reflected gymnasts’ performance (highest competition ever qualified and hours of training in the previous week) and laterality (turning bias and lateralized emotion perception). The implicit achievement, power, and affiliation motives were the main predictors (explicit motives in these same domains represented additional predictors to explore effects of motivational congruence). Perceived failure, perceived autonomy, and type of training represented potential moderators of implicit motives’ association with outcome variables related to sports performance. The study was preregistered at aspredicted.org^1^. Further information and future updates regarding materials, data, and analyses associated with this study can be found at https://osf.io/6rxqj/.

### Procedure

Except for turning bias, which was measured *in situ*, the study was conducted online. Participants were recruited via email after contacting national gymnast clubs and gymnastics federations. We first assessed implicit motives, then administered a chimeric face task, then assessed explicit motives, and finally recorded variables related to gymnastic performance and self-reported autonomy, failure in training, and turning bias. The duration of the online assessment lasted approximately 45 min. The study was conducted online for the most part, except for tuning bias.

### Measures

#### Implicit Motives

The PSE is a standard story-writing measure of implicit motives (Schultheiss and Pang, 2007). It was administered to assess gymnasts’ n Power, n Achievement, and n Affiliation. Participants were asked to write an imaginative story about each of six pictures: ship captain, couple by river, trapeze artists, women in laboratory, boxer, and nightclub scene (see Pang and Schultheiss, 2005). Pictures were presented in a random order, using standard instructions and procedures described in Smith et al. (1992). Each picture was shown on a PC computer screen for 10 s. After viewing the picture, participants had 4 min to write an imaginative story. Stories were coded for motivational imagery (achievement, affiliation, and power) by two independent scorers using the manual of Winter (1994). This scoring system has been validated through experimental arousal of motivational states and demonstrates extensive predictive validity (Winter, 1991; Schultheiss and Pang, 2007). N Power is coded when a person in the story: (1) acts in a strong and powerful manner, (2) controls or manipulates others, (3) tries to persuade or persuades others, (4) seeks help without being asked, (5) wants to impress others, or (6) elicits strong, non-reciprocal emotions in others. n Achievement is coded when: (1) a person’s goals or performance are described with positive adjectives (“excellent,” “good”), (2) goals or achievements are positively evaluated, (3) victory or competition with others are described, (4) someone shows a negative affective response to defeat, or (5) someone performs a unique feat. n Affiliation is coded when a person: (1) experiences positive affects in the context of a relationship, (2) experiences negative affects in regard to the break-up of a relationship, (3) engages in companionable activities with others, or (4) cares about others.

The coders had previously attained greater than 85% reliability with calibration materials prescored by an expert and contained in Winter (1994). They independently coded all stories for motive imagery and were blind to all other participant data during this process. The coders reached satisfactory interrater reliability of *r* = 0.74 for n Achievement, *r* = 0.73 for n Power, and *r* = 0.73 for n Affiliation. The more experienced of the two coders also showed higher sensitivity to motivational imagery in the PSE stories, resulting in higher scores for each of the three motives than the other coder, *t*s(58) > 14.69, *p*s < 0.0001. Thus, the scores of the more experienced coder were used for all further analyses. The coder was blind with regard to all other measurements associated with each participant.

Picture-story exercise protocol length (word count, *M* = 514.07, *SD* = 172.16) correlated with participants’ achievement motive score (*M* = 5.85; *SD* = 2.91), *r* = 0.59, power motive score (*M* = 5.18; *SD* = 2.76), *r* = 0.53, and affiliation motive score (*M* = 6.37; *SD* = 3.03), *r* = 0.61. We therefore corrected the influence of protocol length on motive scores by regressing the word count sum scores from the motive imagery sum scores and converting the residuals to z scores.

#### Performance

Gymnasts were asked to specify the highest competition they ever participated in (higher numbers indicate more advanced competition placement). Participants could choose between World Championships/Olympics (10), German championships (9), other national championships (8), 1st German League (“1. Bundesliga,” 7), 2nd German League (“2. Bundesliga,” 6), 3rd German League (“3. Bundesliga,” 5), upper league (“Oberliga,” 4), association league (“Verbandsliga,” 3), state league (“Landesliga,” 2), and district league (“Bezirksliga,” 1). Participation in higher competition classes requires a better performance or even a listed ranking, assuring that gymnasts participate according to their performance level. In this sample, participants’ performance level range was 1–9 (*M* = 3.93, *SD* = 2.94). Participants also reported how much effort they invested into their physical training (not specifically limited to the training of their gymnastic skills) as the sum of training hours for every day of the last week (e.g., “How many hours did you train last Monday?”).

#### Explicit Motives

Explicit motives were assessed with a cue- and response-matched questionnaire version of the PSE (PSE-Q; Schultheiss et al., 2009), with the subscales achievement as power and affiliation. In the PSE-Q participants were asked to indicate, in a true (1)/false (0) format, what they would do, feel, or want if they were one of the people shown in the same six pictures (ship captain, couple by river, trapeze artists, women in laboratory, boxer, and nightclub scene) as used in the PSE (Pang and Schultheiss, 2005). For every picture, participants had to respond to 15 statements (e.g., “I would try to control the other person” from the power scale or “I would share friendly activities with the other person(s)” from the affiliation scale). Pictures were presented in random order, using standard instructions and procedures as described in Schultheiss et al. (2009). Each scale’s score represents the sum of all endorsed scale-specific items across all pictures. Internal consistency coefficients for the subscales were α = 0.86 for achievement, α = 0.65 for power, and α = 0.67 for affiliation.

#### Turning Bias

Turning bias, as an estimator of lateralized dopaminergic activity (Bracha et al., 1989), was measured in an empty hallway (1.60 m wide, 6.00 m long). The gymnasts were led into the hallway one by one. They were asked to start at a cross marked on the floor. The task was to walk from one marker to the other and then turn around and walk back. The same route should be covered five times, first walking as usual, then walking with the left eye held shut, then with the right eye held shut, then with the right ear held shut, and finally with the left ear held shut. These variations were introduced to provide a reason for having participants walk several times from one marker to the next and to conceal what the measurement was really about. As a consequence, nine turns in total were counted for each participant. The experimenter recorded the total of left turns and right turns. These were later converted into a turning bias quotient according to the formula [(left turns – right turns) × 100]/(right turns + left turns). Negative values reflect increased right turns and positive values reflect increased left turns. Accordingly, positive values stand for stronger right-hemispheric dopamine activity (after coding each left turn as 0 and each right turn as 1, α = 0.69 and split-half-reliability = 0.65).

#### Chimeric Face Task (CFT)

The CFT is a measure to assess emotional-perception laterality (Innes et al., 2016). The version we used depicts chimeric images (i.e., two spliced half faces of the same individual) of faces, with one half expressing happiness and one half a neutral affect. On each trial, two versions of this chimera are shown side by side (i.e., on the left and right half of the screen) – one with the emotion on the left half of the face, one with the emotion on the right half of the face. Pairing (e.g., emotion on left half of the face shown on the left position and emotion on right half of the face shown on the right position, and vice versa) was balanced across blocks, with each block containing an equal number of emotional-left-half chimera being shown on the left side or on the right side of the screen. Participants are required to indicate by key press which of the two chimera versions presented simultaneously appears more emotional to them. The CFT we employed consists of 4 × 4 photographs – half in black and white, half in color – with faces of eight women and eight men. Combined with the position counterbalancing achieved across two blocks, this yielded a total of 32 stimulus pairs. These 32 stimulus pairs were shown once to every participant. Like in the case of turning bias, we calculated a laterality quotient from participants’ key press data on which values could range from −1 (each rating went in favor of the emotional half being on the right side of the face) to +1 (each rating went in favor of the emotional half being on the left side of the face). A positive value thus reflects preferential recognition of emotionality in the left visual field and thus a right hemispheric bias (α = 0.82; split-half-reliability across blocks = 0.82).

#### Perceived Failure

Gymnasts’ perceptions of failure were assessed as a sport-specific incentive for n Achievement, using a self-developed self-report measure including the questions: “Recently I have experienced more failure during my training sessions” (German: “In letzter Zeit erlebe ich im Training vermehrt Misserfolge.”) and “I made little progress during training” (German: “Ich habe im Training nur wenige Fortschritte gemacht.”). Response options on a 7-point Likert scale format ranged from 1 = *not at all applicable* to 7 = *fully applicable* (α = 0.76).

#### Perceived Autonomy

Perceived autonomy was assessed as a sport-specific incentive for n Achievement using the eponymous subscale of the Psychological Need Satisfaction in Exercise Scale (PNSE; Wilson et al., 2006). The PNSE subscale consists of six questions (e.g., “I feel free to exercise in my own way,” “I feel free to make my own exercise program decisions”) with response options on a 7-point Likert scale format ranging from 1 = *not at all applicable* to 7 = *fully applicable* (α = 0.88).

#### Type of Training

To assess the social aspects of training as an incentive for n Affiliation, gymnasts were asked to state whether they usually train alone or together in a group (0 = I always train alone and I train together with others sometimes 1 = I train together with others always).

### Statistical Analysis

All dependent variables were checked for outliers and conformity to a normal distribution was tested using the Kolmogorov–Smirnov test. To investigate associations between implicit motives and indicators of cognitive-behavioral and motor indicators of rightward brain laterality, Pearson correlation analyses were conducted. All correlations retained their approximate size and significance level when we used Spearman rank-order correlations instead of Pearson correlations to ascertain that the observed associations were not due to the influence of extreme values (see Supplementary Table S1 in Supplementary Material). In addition, we conducted moderator analyses to explore possible effects of age, sex, and competition level on the focal associations. To explore the influence of suitable motive incentives, contributing to better sports performance, moderator analyses were run. All statistical analyses were performed using IBM SPSS 21 (Chicago, IL, United States), with *p*-values < 0.05 used as significance threshold.

## Results

**FIGURE 1:**
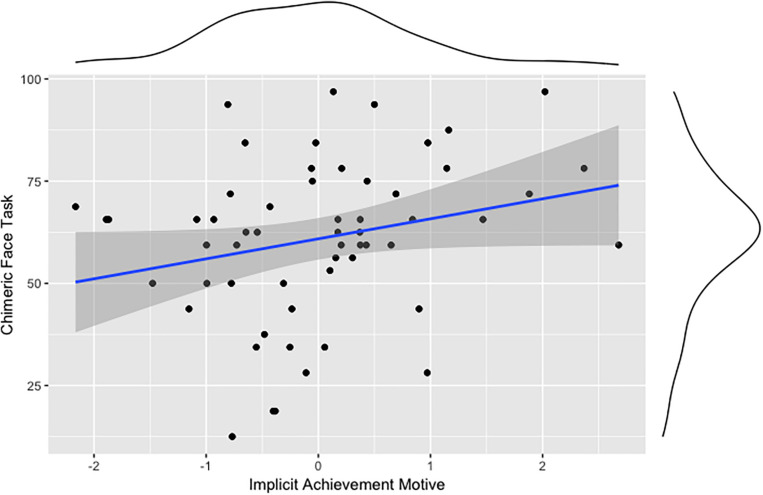
Association between the implicit achievement motive and cognitive-behavioral indicators of rightward brain laterality (CFT).

**FIGURE 2:**
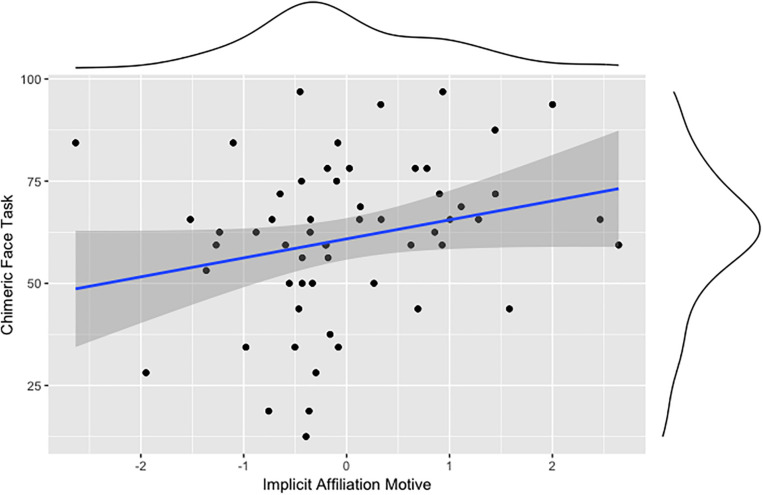
Association between the implicit affiliation motive and cognitive-behavioral indicators of rightward brain laterality (CFT).

Partly in line with our first preregistered hypothesis, implicit motives are associated with cognitive-behavioral indicators of rightward brain laterality. The analysis shows that emotional-perception laterality (CFT) correlates positively with the implicit achievement motive (Figure 1) and the implicit affiliation motive (Figure 2), but not with the implicit power motive. Contrary to expectations, the correlation analyses show that the measure of motor laterality (turning bias) is not associated with any implicit motive.

**FIGURE 3:**
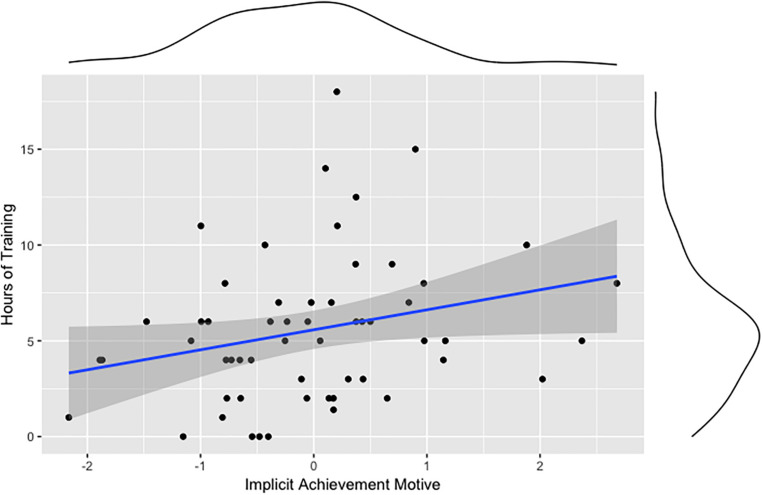
Association between the implicit achievement motive and hours of training.

**FIGURE 4:**
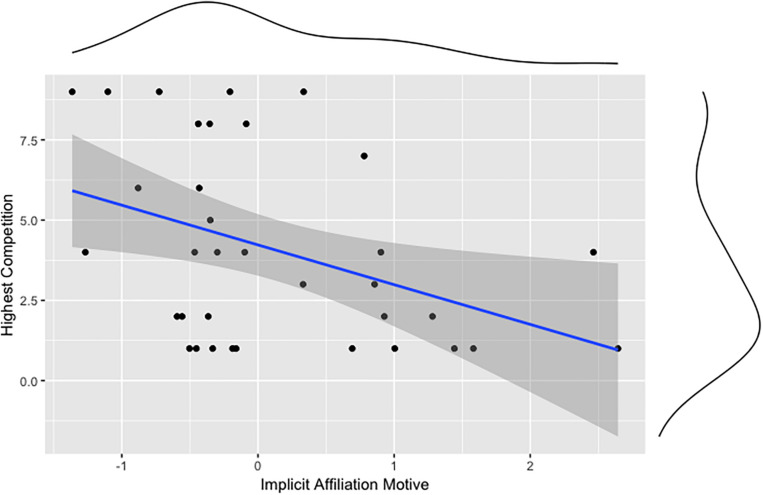
Association between the implicit affiliation motive and the highest competition gymnasts participated in.

**FIGURE 5:**
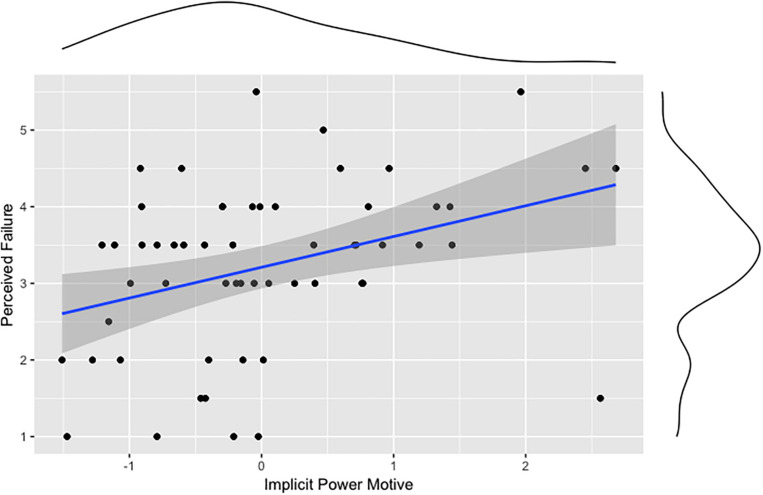
Association between the implicit power motive and perceived failure.

Our second preregistered hypothesis states that implicit motives (achievement, power, affiliation) are associated with sports performance (hours of training, highest competition level). Correlation analyses show that n Achievement correlates positively with the hours of training (Figure 3), but not with the highest competition level. n Affiliation correlates negatively with the highest competition level (Figure 4). No significant correlation was found for n Power and measures of sports performance. But the n Power correlated positively with perceived failure (Figure 5). Table 1 shows all correlations of motive, laterality, and performance measures. We also tested whether sex, age, or athletic ability moderated the relationships between implicit motives and criterion variables depicted in Figure 1 through 5, but found no evidence for this being the case (*p*s > 0.05).

**TABLE 1:** Pearson correlations (Ns) of motives, laterality, performance, and moderators.

	1	2	3	4	5	6	7	8	9	10	11	12	13
1 Implicit achievement motive		−0.22*(60)	0.20(60)	0.15(57)	−0.11(57)	−0.08(57)	0.15(33)	0.25*(57)	−0.14(35)	0.27*(56)	0.02(57)	0.15(57)	0.03(56)
2 Implicit power motive			−0.16(60)	−0.01(57)	0.40**(57)	0.11(57)	0.07(33)	−0.22(57)	0.02(35)	0.00(56)	0.36**(57)	−0.01(57)	−0.21(56)
3 Implicit affiliation motive				0.04(57)	0.06(57)	0.25*(57)	−0.24(33)	0.24*(57)	−0.40**(35)	−0.07(56)	−0.04(57)	0.01(57)	0.10(56)
4 Explicit achievement motive					0.36**(63)	0.33**(63)	0.05(32)	0.18(63)	−0.11(40)	−0.12(62)	0.18(63)	0.08(63)	−0.13(62)
5 Explicit power motive						0.45**(63)	−0.14(32)	0.03(63)	−0.21(40)	−0.15(62)	0.12(63)	0.05(63)	−0.16(62)
6 Explicit affiliation motive							−0.05(32)	0.17(63)	−0.20(40)	−0.32**(62)	−0.05(63)	−0.16(63)	−0.02(62)
7 Turning bias								−0.26(33)	0.09(21)	0.31*(31)	0.00(32)	−0.01(32)	0.25(31)
8 Chimeric face task									0.26*(42)	0.03(65)	−0.18(67)	−0.08(67)	0.03(66)
9 Highest competition										0.04(41)	−0.29*(42)	−0.09(42)	−0.20(41)
10 Hours of training											0.02(65)	0.07(65)	0.12(65)
11 Failure												0.10(67)	0.03(66)
12 Autonomy													0.05(66)
13 Training together/alone													
*M*	7.18	5.83	8.75	17.89	16.83	9.67	65.36	60.53	3.93	5.69	3.24	4.84	0.56
*SD*	3.73	3.31	4.41	4.44	5.03	3.30	24.73	19.54	2.94	3.76	1.16	1.06	0.50

While the descriptive statistics provided for PSE motive measures are based on raw scores, all correlations involving PSE motive measures were based on word-count-residualized motive scores. *p < 0.05, **p < 0.01, one-tailed.

To test our third preregistered hypothesis – implicit motives contribute to better sports performance given the presence of suitable achievement and affiliation incentives – we conducted moderator analyses. Results indicate that neither perceived control nor failure moderated the relationship between the implicit achievement motive and hours of training, *b* = −0.116, 95% CI [−1.511, 1.279], *t* = −0.167, *n.s* and *b* = 0.426, 95% CI [−0.696, 1.549], *t* = 0.762, *n.s.*, respectively. An additional moderator analysis was conducted to examine whether training together vs. alone moderates the relationship between the implicit affiliation motive and hours invested in training. The results show that this is not the case, *b* = 0.988, 95% CI [−1.083, 3.060], *t* = 0.957, *n.s.* Thus, our third hypothesis was not supported.

We also explored to what extent congruence between implicit motives (PSE) and self-attributed motives (PSE-Q) contributes to a better sports performance. Results indicate that the congruence between implicit motives and self-attributed motives does not contribute to better sports performance when assessed via hours of training nor highest competition, but does when assessed via the power motive. In brief, the implicit power motive was positively associated with hours of trainings in individuals low in explicit power motivation but not in participants with high or medium level of this variable. Table 2 shows the results of the exploratory analysis.

**TABLE 2:** Regression analyses testing moderator effects of explicit motives on the association between implicit motives and sports performance measures.

Measures of performance	*t*	*p*	β	*F*	*df*	*p*	*adj.R*
**Hours of training**							
Overall model				2.00	3, 52	0.13	0.05
Implicit achievement motive	1.49	0.144	1.04				
Explicit achievement motive	–0.76	0.449	–0.10				
Interaction	–1.10	0.275	–0.78				
**Highest competition**							
Overall model				1.21	3, 31	0.36	0.01
Implicit achievement motive	–1.66	0.107	–1.74				
Explicit achievement motive	–0.79	0.436	–0.14				
Interaction	1.57	0.127	1.65				
**Hours of training**							
Overall model				2.42	3, 52	0.08	0.07
Implicit affiliation motive	–1.15	0.257	–0.60				
Explicit affiliation motive	–2.14	0.037	–0.29				
Interaction	1.20	0.237	0.62				
**Highest competition**							
Overall model				3.82	3, 31	0.02*	0.20
Implicit affiliation motive	–1.70	0.100	–1.20				
Explicit affiliation motive	–1.13	0.267	–0.19				
Interaction	1.23	0.227	0.86				
**Hours of training**							
Overall model				1.98	3, 52	0.13	0.05
Implicit power motive	2.28	0.027*	1.32				
Explicit power motive	–1.28	0.207	–0.19				
Interaction	–2.25	0.028*	–1.27				
**Highest competition**							
Overall model				1.13	3, 31	0.35	0.01
Implicit power motive	0.97	0.338	0.74				
Explicit power motive	–1.82	0.078	–0.34				
Interaction	–0.89	0.378	–0.67				

All variables were mean centered before analysis. *p < 0.05.

## Discussion

In this preregistered study, we explored whether implicit motives are associated with indicators of right-hemispheric functions. We also examined whether implicit motives are linked to sports performance (hours of training, highest competition level ever participated in) and whether this link is moderated by motivational incentives (failure, autonomy, social aspects of training).

Before we discuss our findings in detail, we want to point out that due to the small sample size all findings should be seen as strictly tentative and not conclusive. The low statistical power from small sample sizes can lead to false positive results, incorrectly rejecting the null hypothesis, or to false negative results, incorrectly accepting the null hypothesis (see Button et al., 2013). Therefore, all results discussed in this section should be seen as preliminary only and interpreted with caution.

Our first preregistered hypothesis states that implicit motives are associated with rightward laterality. Our analyses partly support the preregistered hypothesis for indicators of perceptual-emotional laterality assessed using the CFT. The implicit affiliation motive and the implicit achievement motive both correlated positively with the CFT, indicating right hemispheric bias of these motives, at least with regard to the specific function of evaluating affective social signals. This means that for individuals high in either motive, the face appears more emotional with the emotional expression in the left visual field. These results confirm previous findings, showing that people high in n Affiliation show an increased sensitivity to faces (Atkinson and Walker, 1958; Schultheiss and Hale, 2007). Contrary to our hypothesis, however, we did not find a significant correlation between the implicit power motive and perceptual-emotional indicators of a right-hemispheric bias. One explanation for this could be that associations of the implicit power motive with relevant criteria often depends on moderators such as activity inhibition (see, for instance, Rösch et al., 2013, and Schultheiss, 2018). Another reason may be low statistical power contributing to a false negative result in this case.

Therefore, only the correlations of the CFT with n Achievement and n Affiliation confirm our preregistered hypothesis for perceptual laterality to some extent, whereas turning bias as a measure of motor laterality showed no associations with implicit motives. This suggests that the link between motives and brain laterality may be easier to demonstrate for perceptual-emotional functions than for motor movement. But again, our ability to detect any systematic effect either for or against our hypothesis was severely limited by the small sample size for this particular set of analyses.

Our second preregistered hypothesis states that implicit motives contribute to sports performance. In line with this hypothesis, correlation analyses showed that n Achievement predicted longer training hours. In contrast, the explicit achievement motive did not correlate with training hours. Gröpel et al.(2016, Study 2) found similar results: The implicit achievement motive predicted sports participation, but the explicit achievement motive did not. These observations are consistent with McClelland’s (1980, 1989) suggestion that implicit motives predict long-term trends in behavior better than explicit motives. In contrast to n Achievement, the implicit needs for affiliation and power motive did not correlate with training hours. One reason for this could be that according to Krug and Kuhl (2006) a high achievement motive is particularly critical in sports such as gymnastics that are based on technical elements. In order to achieve excellent performance results, gymnasts have to improve constantly and work on their individual movement patterns and techniques. In contrast, according to Krug and Kuhl the power motive is more important in sports emphasizing interactions and fighting. The affiliation motive seems to play a less important role in sports except for team sports, where it may be relevant for team cohesion.

The implicit achievement motive does not correlate with the highest competition ever participated in. One might assume that the implicit achievement motive would also correlate with competition, as a high number of training hours might lead to higher competition rankings. In our study, we could not confirm this link between hours of training and highest competition ever participated in, perhaps because our measure of training intensity did not adequately represent the overall effort athletes invested in their skills. Another reason for the lack of association between competition rankings and training hours or n Achievement may be that athletes’ psychological and physical daily form can exert a strong effect on her or his performance. There are many variables that may influence performance during a competition more than training hours, such as emotions, especially stress, and anxiety (Jokela and Hanin, 1999; Craft et al., 2003; Hanin, 2007). Unexpectedly, the implicit affiliation motive correlated negatively with the highest competition ever participated in. Perhaps this is due to the rivalry that competitions are characterized by, as gymnastics is an individual sport and not a team sport. In individual competitions, former teammates and training colleagues become rivals. In such situations, friendly relationships between gymnasts are anathema for the goal of winning. Gymnasts high in n Affiliation therefore likely experience competitive situations as aversive and prefer situations in which their performance can be based on collaboration (Atkinson and O’Connor, 1966).

In addition to the limitations we already highlighted, this study has several other limitations. Participants were of a very special and homogeneous competitive sport sample, for which a larger pool of test persons was not available. Nevertheless, we see value in research on real-world aspects of behavior, such as sports, and highly select samples such as ours, as both may shed light on how motivational dispositions affect consequential behavior. This is also the reason why we decided to publish this research, despite its small sample size. We hope that as others conduct similar studies and publish them (regardless of whether findings confirm hypotheses or not), this will enable future researchers to meta-analyze the results from several such studies one day and thereby help to tease out robust and substantial associations across studies. Another limitation of our research was the lack of specificity of the PSE we used. Schultheiss and Pang (2007) argued that researchers should aim at choosing picture cues that resemble the situation for which one wants to predict behavioral outcomes with motive measurements. This was not the case in our study, because we had opted instead for better comparability of PSE scores with earlier studies and used the Pang and Schultheiss’s (2005) standard broadband measure. Future studies on gymnasts and other athletes and their sports performance should try to employ more custom-tailored PSE showing, for instance, pictures of athletes in various sports contexts. Further limitations relate to the self-reported nature of many training and performance measures as well as our inability to provide a measure of interrater reliability of the turning bias, due to the fact that turns were recorded *in situ* and not from video recordings.

## Conclusion

In conclusion, our exploratory results suggest that there seems to be a link between implicit motives (achievement and affiliation) and perceptual laterality. Moreover, people high in implicit affiliation motive might not like competitive situations with teammates acting as rivals and therefore show lower performance results concerning competitive situations. Training interventions specialized to reduce the stress and struggle during competitions could help gymnasts high in the implicit affiliation motive to better regulate their focus and concentration. A high implicit achievement motive can predict how often people engage in sports. People high in achievement motive experience inherent joy from the exercise itself and from the mastery of challenging exercises. Therefore, fun and enjoyment could be seen as a justification for engaging in sports (see also Gröpel et al., 2016). The incentives (autonomy, failure) seem to be less relevant, at least at the self-report level. These findings suggest that making sports enjoyable may contribute to higher sports participation.

## Data Availability Statement

The original contributions presented in the study are publicly available. This data can be found here: https://osf.io/6rxqj/.

## Ethics Statement

This study was carried out in accordance with the recommendations of the American Psychological Association’s Ethics Code, with written informed consent from all subjects. All subjects gave written informed consent in accordance with the Declaration of Helsinki.

## Author Contributions

OS and L-MS designed the study, coded all picture stories and conducted all statistical analyses, and wrote the manuscript. L-MS recruited, and tested all participants. Both authors read and approved of the final manuscript.

## Conflict of Interest

The authors declare that the research was conducted in the absence of any commercial or financial relationships that could be construed as a potential conflict of interest.

## Footnotes

1 https://aspredicted.org/ix72r.pdf
