# Supplementary material for: Implicit Motives, Laterality, Sports Participation and Competition in Gymnasts
Source: Front Psychol. 2020 May 26;11:900. doi: 10.3389/fpsyg.2020.00900 (PMC7265554; doi:10.3389/fpsyg.2020.00900)
Supplement: Supplementary file 1 [file Table_1.pdf]

Supplement for

Schütz, L.-M., & Schultheiss, O. C. (2020). Implicit motives, laterality, sports participation and competition in gymnasts. *Frontiers in Psychology*.

Table S1.

Spearman rank-order correlations (Ns) of motive, laterality, performance and moderator variables

	1	2	3	4	5	6	7	8	9	10	11	12	13
1 implicit achievement motive		-.16(60)	.19(60)	.22*(57)	-.02(57)	-.07(57)	.10(33)	.27*(57)	-.15(35)	.31*(56)	.08(57)	.20(57)	.00(56)
2 implicit power motive			-.17(60)	.01(57)	.39**(57)	.07(57)	.03(33)	-.21(57)	.11(35)	.09(56)	.36**(57)	-.03(57)	-.19(56)
3 implicit affiliation motive				.16(57)	.10(57)	.30*(57)	-.14(33)	.29*(57)	-.39*(35)	-.06(56)	-.06(57)	.15(57)	.08(56)
4 explicit achievement motive					.38**(63)	.29*(63)	.08(32)	.14(63)	-.10(40)	-.12(62)	.20(63)	.08(63)	-.11(62)
5 explicit power motive						.47**(63)	-.12(32)	.05(63)	-.12(40)	-.09(62)	.08(63)	.06(63)	-.15(62)
6 explicit affiliation motive							-.00(32)	.17(63)	-.18(40)	-.25*(62)	-.04(63)	-.12(63)	-.02(62)
7 Turning Bias								-.28(33)	.10(21)	.27(31)	.01(32)	-.19(32)	.24(31)
8 Chimeric Face Task									.25(42)	-.01(65)	-.17(67)	-.07(67)	.02(66)
9 highest competition										.02(41)	-.31*(42)	-.02(42)	-.17(41)
10 hours of training											.05(65)	.08(65)	.13(65)
11 failure												-.01(67)	.02(66)
12 autonomy													.01(66)
13 training together/alone													
M	7.18	5.83	8.75	17.89	16.83	9.67	65.36	60.53	3.93	5.69	3.24	4.84	0.56
SD	3.73	3.31	4.41	4.44	5.03	3.30	24.73	19.54	2.94	3.76	1.16	1.06	0.50

Note. While the descriptive statistics provided for PSE motive measures are based on raw scores, all correlations involving PSE motive measures were based on word-count-residualized motive scores. *$p < .05$, **$p < .01$, one-tailed.
